# On Vaccination and Variolous Diseases

**Published:** 1864-07

**Authors:** O. Kratz

**Affiliations:** Surgeon P. A. C. S.


Art. IV.—*On Vaccination and Variolous Diseases.* By O. Kratz, Surgeon P. A. C. S.

The following remarks on vaccination and variolous diseases have been suggested to me from my own experience. Spurious vaccine and its deleterious influences on vaccination seem to me worthy of further investigation, as a subject hitherto not sufficiently appreciated.

In order to define my stand-point clearly, let me state, at the outset, that Liebig's Theory of Fermentation is, till now, the best to explain the phenomena of vaccination, and that all the anomalies occurring, may be best elucidated by this hypothesis. At the same time I do not feel warranted in subscribing blindly to it; but I think we have not any better as yet, and I simply adopt it in the same manner as I would have to conform to the atomic theory in treating about chemistry.

In vaccinating a subject we introduce, then, the yeast, the virus, into the circulation, to produce the fermentation and its result, the scab. If there is a certain substance in the system for the virus to re-act upon, the scab will be formed and the subject is vaccinated. If, on the contrary, this substance is entirely deficient or modified in some way or other, no scab or an imperfect one will be the result.

With very few exceptions, the good vaccine matter will produce a normal scab on the subject never vaccinated before. The re-acting matter in the system is eliminated, and by this process the liability to the infection of small-pox rendered impossible or greatly diminished. Possibly, in a long interval of time, this matter may be re-organized in the body, but never to its original state. I say never, because such a subject may be attacked by varioloides, but never by variola.

If this re-acting matter is re-organized as nearly as possible to its original state, the second vaccination will produce a scab also, but never a perfect one. It may be perfect for the protection of the individual on whom it appears, but it offers no guarantee for re-vaccination on other subjects. I call this a pseudo-scab. This scab may yet retain much of the original fermenting property, but not the same as the genuine. If this virus is used again for the re-vaccination of an individual, its product will offer again less guarantees of being protective. Continuing in this manner, the scab will finally contain nothing but common suppurative matter—pus—as if taken from any other suppurating place in the body. The form of the scab itself will be more or less modified and deviating from its normal structure.

As I have no book of reference on the subject, I quote the following from memory:

Some years ago, the Academy of Medicine in Paris investigated very closely the fact, if syphilis could be transferred by vaccination. The result obtained by repeated and most direct experiments was, that it could not be communicated by transmission of genuine vaccine virus from one individual to another, affected with syphilis in any of its stages. Liebig's explanatory theory of fermentation holds good here. The vaccine virus will re-act only on certain substances in the system and ignore others entirely. We know, at the same time, that matter from a syphilitic suppurating surface will reproduce syphilitic symptoms on another subject. I have had occasion to observe several well-defined cases of Rupia syphilitica produced solely by vaccination. Other respectable surgeons, worthy of implicit belief from their scientific attainments, have noticed the same and similar facts repeatedly.

These subjects had never syphilis before, otherwise the inference would be doubtful or worthless altogether. Therefore, instead of having had vaccine virus inserted, they had been inoculated with syphilis. I have seen one case where the product of the vaccination was surpetigo rodeus, a frightful disease of, I believe, a cancerous character. Some cases had herpes excedens as the result of vaccination on their arm.

The syphilitic cases had been treated for other cutaneous diseases without any material amelioration. The mercurial treatment removed the symptoms at once. I have never seen anomalous results from vaccination if the following precaution was strictly adhered to: The vaccine matter used was taken from a healthy infant, never vaccinated before.

The indiscriminate vaccination and re-vaccination from arm to arm has been, in my opinion, the principal cause of the deterioration of the vaccine virus, and of producing cutaneous diseases from vaccination. A second cause may be found in the fact, that the virus used is old and too rarely regenerated by passing it through the cow. Cow virus will fail only 1 in 100; good common virus will fail 3 in 100, if I remember right.

